# Informational Power and Perceived Collective Benefit Affecting the Users’ Preference for a Mobile Technology: Evidences From a Survey Study

**DOI:** 10.3389/fpsyg.2018.00898

**Published:** 2018-06-06

**Authors:** Stefania Fantinelli, Michela Cortini

**Affiliations:** Department of Psychological, Health, and Territorial Sciences, Università degli Studi “G. d’Annunzio” Chieti-Pescara, Chieti, Italy

**Keywords:** mobile technologies, persuasive technologies, collective benefit, attitude and behavior change, informational power

## Abstract

This study takes place from the idea that the personal usage of mobile technologies can bring positive outcomes to the user and to their society in an indirect way. Technologies studied in this work are defined as persuasive technologies (Fogg, 1997) because they are intentionally designed to modify the users’ attitude or behavior. This research is aimed to evaluate if the intention to use the application can be influenced by positive attitudes toward technology, by the persuasive power of the application and by the perceived fun. Participants (*N* = 118; *M* = 55; *F* = 63; mean age = 27.4; range age = 15–69) filled in an online questionnaire that was partly based on the Media and Technology Usage and Attitude Scale (MTUAS – Rosen et al., 2013). An additional eight items were added to the scale, aimed at evaluating participants’ technophobia, technophilia, perceived technology pervasiveness and perceived persuasive power of technology. By using linear regression analysis, it was found that the application’s informational power and the perceived entertainment positively influenced the usage intention. Another interesting result, obtained through ANOVA, concerns a generational difference: baby boomers tended to trust more the fact that the single individual action through the application can have an effective impact on the environment. These results represent a basis for future in-depth investigations about socially relevant use of the ICT.

## Introduction

Pervasiveness, ubiquity, interconnection, speed and innovation are all terms that often accompany the concept of information and communication technologies. They also describe the characteristics of today’s modern society. There are concerns in different subject areas about the influence and the changes that technology is inevitably contributing to society; the development of this work is born by an interest in the effects that technological progress and its inevitable foray into our lives are producing in society.

Technological immersion also concerns the workplace environment with recent new trends: the personal use of social media can be described as both opportunities and hindrance for employees with regards to their work life balance (Cortini, 2009; Cortini and Scaratti, 2011). On the other hand, social media can be a source for employers in the knowledge management process (Fantinelli, 2017).

In the current paper our attention is directed toward mobile technologies used by individuals on a daily basis, from the simple cell phones to smartphones, from tablets to smartwatches or other wearable devices.

The aim was to investigate the possible positive effects that technological advancements can have on individuals and society, through the simple and daily use of mobile devices. Such devices have been defined as *persuasive technologies*, meaning, technologies intentionally designed to modify a users’ attitude and/or behavior (Fogg, 1997). Fogg initiated this area of study inventing the neologism *captology*, Computers As Persuasive Technologies (Fogg, 1997). Persuasion, being purely a communicative act directed to facilitate a voluntary behavioral or attitudinal change and not to manipulate or coerce.

The intent was to provide evidences relating to the use of a persuasive technology for an altruistic purpose; the persuasive power could promote social benefits through the use and action of the individual.

Two mock persuasive applications were presented to the participants: the first application was related to a healthy lifestyle (fantasy name PosiLive); the persuasive intent in this app was to modify the users attitude and/or behavior about sport activities and their perceived benefits. The second application was called ViviEco: which related to promoting a sustainable lifestyle. The persuasive purpose in this case was to modify daily habits with regards to respectful choices about the environment, so the perceived benefits are directed to both the single user and society.

The main dependent variable is the user’s intention to use the applications, considering the influence of the applications’ persuasive power, perceived fun and informative power. Only the perceived impact for the application ViviEco was measured in this study: it was the users’ perception about the effectiveness of their own actions on the environment.

Results suggested that the intention to use the application was positively affected by both the informational power and the perceived fun. This result is in line with a previous research study by Hur et al. (2017) that was based on Technology Acceptance Model (Davis, 1989). With regards to the perceived impact, there was a generational effect: baby boomers manifested a higher awareness than younger participants about their own actions through the application.

This latter evidence confirms some opposite positions to the digital natives definition (Bennett et al., 2008; Helsper and Eynon, 2010), said in different terms, according to our results participants in the eldest rank had more consciousness of technology’s benefit than younger participants.

At the best of our knowledge this is the first study aimed at evaluating the so-called informational power as a determinant variable affecting the intention to use a technology. In addition, as far as we are concerned the perceived impact should be a variable that is worth taken into consideration when designing new persuasive technology.

The theoretical framework of this study was partly constituted by Fogg’s (1997) theory about persuasive technologies, together with the planned behavior theory (Fishbein and Ajzen, 1975), which contributes to the comprehension of the Technology Acceptance Model (Davis, 1989). The planned behavior theory is useful to explain the link between attitudes and behavior, referring also to those behaviors which are not under total individual control, such as common habits. It has been adopted a quantitative methodology: it has been elaborated an *ad hoc* questionnaire that was published through Google Forms and then the collected data were analyzed with the help of the statistical software package SPSS 21.0 by IBM.

### Literature Review

Fogg has initiated a new area of research that fits across psychology, sociology and technology; in particular, he is interested in the study development and implementation of persuasive technologies. His studies and those conducted by other researchers in the same field of research, explore the interaction between user and technological devices from several points of view: from the design of interactive technologies created with the intention of encouraging a change in attitude or behavior in the user; but also from a cognitive and psychosocial point of view, investigating the reactions of users and the impact on society. Fogg also developed new applications of persuasive communication to technology (Fogg, 2003). Persuasive technologies are designed according to several principles, one of the most relevant and implemented is contextual communication, which is a feature that provides information or messages in a context sensitive way; indeed just-in-time messaging can motivate behavior changes (Romão, 2016). Furthermore, Fishbein and Ajzen (1975) work about attitudes and behavior changes is crucial in order to explain the aim of persuasive technologies. They elaborated on the most famous theory in the field of attitudes: the *theory of reasoned action*, which will later be revised. They also expanded and renamed the *theory of planned behavior* (1991). The theory of reasoned action explains what are the predictors of behavior: the first being the intention to implement a given behavior; the intention is in turn predicted by the attitude toward the behavior and by subjective norms, defined as beliefs that significant others have with respect to the behavior (Ajzen, 1991). Finally, the attitude is determined by behavioral beliefs and subjective norms and are determined by normative beliefs (Fishbein and Ajzen, 1975).

The theory of planned behavior also takes into account the behaviors that are not under the total control of the individual; therefore it was added a new variable to the previous model, that is the subjective perceived control that has a direct influence on both the intention and behavior.

Sundar’s (2012) contribution to the SAGE Handbook of Persuasion is useful to understand how technology can persuade; it specified that technology is not just a channel or a medium to communicate messages, but it can be the actor of persuasion as well. According to past studies about Computers As Social Actors (CASA), individuals tend to interact with computers in the same way they interact with other humans (Reeves and Nass, 1996). Furthermore, technology can be perceived as more or less credible, just as humans.

When comparing real persuasion to the digital one, it is possible to highlight other features of persuasive technologies. For example, the informational power, the persuasive power and enjoyment, that have already been studied in past studies.

In persuasive communication, the subject of the message is one of the main variables which can determine the success of the persuasion. McGuire (1968) elaborated on the information processing theory in order to explain how important was the relevance and the comprehension of the persuasive message.

Informational power has been particularly studied in the field of health technologies, providing information through technology can foster the system’s credibility and in turn affect patient’s commitment and self-efficacy (Fogg, 2003). Moreover, the dissemination of health information can function as a motivational boost and incentive to patient’s engagement in their own health (Graffigna et al., 2017).

Persuasive power is the degree to which the persuasive intent had a successful outcome, that is, a change in attitude or behavior (Cavazza, 2009). As previously mentioned, a persuasive effect can be applied to both humans and computers.

In order to make apps fun or entertaining for participants, they should take on a digital game concept, with the challenge of reaching a goal. Past studies have demonstrated that a users’ active interaction with a digital game can increase their motivation and engagement (Lieberman, 1982; Sundar and Kim, 2005). Moreover, a digital game can be an engaging environment for many different situations, such as: attitude or behavior changes, learning and skills development (Lieberman, 2015). According to the self-determination theory (Deci and Ryan, 2002), there could be intrinsic and extrinsic motivations to boost the user’s engagement. The most powerful intrinsic needs are for autonomy, competence and social connectedness. Contrastingly extrinsic motivations can motivate users just for a superficial engagement (Lieberman, 2015).

Informational power, persuasive power and the perceived fun are the main variables investigated in this present study: the study explores persuasive technologies with a focus on the social implications derived from the personal use of these technologies. Social implications can defined as the collective benefit which comes from the user’s impact on the environment suggested by the technological application. This choice has been driven by the fact that environmental theme is based on objective universally recognized and shared data. Although with cultural differences, dictated by climate and social conditions of each country, global initiatives are still ongoing in order to promote pro-environmental behaviors and attitudes in individuals and communities.

In 2008, Australia held a workshop titled *Pervasive persuasive technology and environmental sustainability.* Is was the first relevant research conferences carried out with a similar theme to this paper. There were three main objectives and topics covered: overcome the simple informational and communicative function of new technologies to make them as incentives to action and change; avoid that pervasive technologies would become invasive and adopt an ecological approach to assess their impact and implementation; finally, evaluate the use of environmental sensors designed to communicate needs and any environmental problems directly to individuals (Foth et al., 2008).

Furthermore Kimura and Nakajima (2011) research premise was the distinction between collectivist and individualist societies: many researches carried out in the persuasive technologies field were generally related to individualist populations (especially Americans). The work of Kimura and Nakajima (2011) held in Japan, which is typically a collectivist country (Kimura and Nakajima, 2011). Part of their research was to evaluate the effectiveness of various persuasive techniques by creating a gamified application^1^, those that produced the greatest impact were the mutual control and the combined use of positive and negative feedback. This study introduces a central variable in the acceptance and usage of persuasive technologies, namely the tendency to collectivism or individualism, but it again proposes a purely playful mode of fruition.

### Research Questions and Hypotheses

The empirical part of this research was built with the intention to further explore and provide evidence relating to the use of persuasive technology for an altruistic purpose; in this way the persuasive power could promote social interests through the individuals use and action of technology. The research questions from which the work started were as follows:

(1) Do users perceive the impact of persuasive technologies that have a social aspect?
The questions asks if the users’ belief related to the personal usage of a persuasive technology could have an actual impact on society, performing pro-environmental behaviors.
(2) Are users more interested in using persuasive technologies for a collective or an individual benefit?
The literature, thus far is mostly focused on the benefits which the single user can obtain, deriving from the attitude or behavior change (Baron and Kenny, 1986; Lenert et al., 2004; Obermayer et al., 2004). The collective benefit is a fairly new research question which this study hopes to answer.
(3) What are the main variables that influence the users’ attitude toward persuasive technologies?
(4) What are the main variables that influence the usage behavior of persuasive technologies?

Past theoretical models and studies have defined some principles to answer the two last questions. According to the Technology Acceptance Model – TAM (Davis, 1989), the perceived ease of use and the perceived usefulness are the two main variables that determine the attitude toward technology, and in turn, the intention to use that technology. For example, when applying the Technology Acceptance Model Godoe and Johansen (2012) found that individuals more optimistic toward technology perceived the specific technology easier to use and more useful.

Another relevant aspect that can affect the intention to use the technology is the persuasive power of a specific technology, that is the success of the persuasion intent. Fogg (2003) provided a detailed description of different principles able to enhance the probability of persuasion; furthermore, according to Lieberman (2015), the gamification effect can positively affect the user’s engagement. Therefore the following was hypothesized:

(1) The intention to use the application in the future is influenced by:
(1a) The positive attitudes toward technology;
(1b) The persuasive power of the application;
(1c) The perceived fun.
The formulation of the following hypotheses were inspired by McGuire’s (1968) theoretical model regarding persuasive communication, where the comprehension and the information of the message can be determinants, but they are not the only variables able to determine the success of a persuasive communication.
(2) The persuasive power can have a mediating role in the relationship between the informational power and the intention to use an application.
(3) The persuasive power can have a mediating role in the relationship between perceived enjoyment and tendency to use an application.

The last hypothesis is related to the perceived impact of the application ViviEco. According to Stern (2000), there is often a lack of correspondence between good intentions toward the environment and the effective behavior. For this reason, we imagine that the awareness of performing good actions for the environment, suggested by the application, can affect the users persuasive intention of the application itself. So the hypothesis was as follow:

(4) The perceived impact of the application ViviEco influences its persuasive power.

### Research Methodology

The research conforms to the provisions of the Declaration of Helsinki (World Medical Association, 2000), and all ethical guidelines were followed as required for conducting human research, including adherence to the legal requirements of the study countries. According to the Italian Association of Psychology (AIP), at the time of the current study, it was available a very general document whose guidelines we have followed. We did not ask for a written consent since it was an online questionnaire on voluntary basis.

The online questionnaire was generated by implementing some recommendations reported by Vicente and Reis (2010) and based on empirical research focused on the comparison between traditional and online questionnaires. For example, it was not included a progress indicator because past empirical evidences (Vicente and Reis, 2010) did not found benefit or advantage for its presence. A traditional graphical presentation was used without inserting special fonts, arrows or graphics that would have enriched the fruition because some studies have not found any advantage over the classical text mode, by contrast, it could serve as a distraction (Deutskens et al., 2004). A limited number of questions were prepared, to avoid dropouts during completion, there is a total of 58 items.

### Measures

The online questionnaire was organized into five sections: the first was dedicated to demographics variables, such as age, sex, and education. The second section evaluated the technology usage and the third section evaluated technology attitudes. The fourth section was dedicated to the introduction of the applications: ViviEco and PosiLive were described and participants were asked to imagine using one of them. The last section was focused on the application acceptance.

Five subscales obtained from the Media and Technology Usage and Attitude Scale developed by Rosen et al., 2013 (MTUAS) were used; the complete instrument had a total of 60 items and 15 subscales overall: 11 of the subscales concerned recognizing the activities and possible uses with different technological devices (cell phone, computer, television, online activities in general, activity on social networks) and the remaining 4 subscales were related to the recognition of the positive and negative attitudes toward specific activities (finding information online, have access to the Internet, keeping up with technological innovations, feeling dependent on technology, believing that technology is the origin of many solutions to current problems or on the contrary, the origin of many hardships).

Rosen et al. (2013) analyzed results from a sample of 942 subjects in order to validate the scale. Their analyses confirmed that all the subscales were internally and externally valid and reliable, so they could be used together or individually (Rosen et al., 2013). The same response scale proposed by Rosen et al. (2013) was used in this study for the items relating to smartphone usage and gaming habits: a 10-point Likert scale was used to indicate the frequency of the target behavior (never, once a month, several times a month, once a week, several times a week, once a day, several times a day, once an hour, several times in an hour, continuously). The choice to use the Likert scale is in line with the Boase and Ling who verified the validity of the measures used to detect frequency of use of smartphones: the most widely used mode was represented by self-report measures, participants were asked to express an estimate frequency of use of their smartphone (Boase and Ling, 2013). An average score was then calculated.

The subscales used in the questionnaire were as follows:

(1) Smartphone usage: nine items that assess the frequency of certain actions with smartphones, such as reading emails, use of Internet browsers, surfing the Internet, listening to music, taking photos, checking the news, recording videos, using applications and searching information. For example, a typical item was: “please indicate how often you do each of the following activities on your mobile phone: search for information with a mobile phone.”
(2) Video games: in the Rosen et al. (2013) scale, this subscale consisted of three items. It was decided to break down the questions to make them clearer, thus obtaining nine items. In particular, the aim was to investigate the frequency of the following activities: play alone on the computer, play alone on video game consoles, play alone on the smartphone, play with other people on the computer, play with other people on consoles, play with other people on the smartphone, play online with other people on the computer, play online with other people on consoles, play online with other people on the smartphone. The following is an example of a question item: “How often do you do each of the following activities? Play games on a computer, video game console or smartphone by yourself.” For the attitudes subscales, the same response scale used in MTUAS was used: a 5-point Likert scale that represents a continuum from strongly disagree position (equivalent to the value of 1) to strongly agree position (equivalent to the value of 5), with the central value that expresses the neutral position “neither agree nor disagree” (equivalent to the value of 3).
(3) Positive attitudes toward technology: six items to evaluate the attitudes through the participants’ opinions on the importance of finding any information online, on free access to the Internet, on being in step with the latest innovations, on the fact that technology can provide solutions to many problems, on the idea that technology makes everything possible and on the feeling that technology can enhance personal skills. For example an item was: “I think it is important to keep up with the latest trends in technology.”
(4) Negative attitudes toward technology: three items that detect negative opinions on some assumptions toward technology. The fact that new technologies contribute to wasting time, making life more complicated and encouraging social isolation. For example an item was: “New technology makes people more isolated.”
(5) Anxiety/dependence on technology: three items designed to measure opinions on several characteristics of technology dependency. Being anxious when deprived of the smartphone, when there is no internet connection available and declaring dependency on technological devices. For example: “I get anxious when I don’t have my cell phone.”

Additional items derived from literature reviews and from previous research studies concerning persuasive technology were added. The response scale was a 5-point Likert scale which was used previously for the attitudes subscales. The items were classified as follows:

(1) Technophobia or critical attitude toward technology: three items that detect the opinions related to the idea that technology is ruining the younger generation, which is depriving people of their freedom and the idea that special training should be required in order to fully exploit the advantages offered by the technology. For example an item was: “Technology is ruining new generations.”
(2) Technophilia or euphoric attitude toward technology: three items that unlike the previous items enhance the optimism of technological development. The aim was to collect opinions about the fact that the technology can offer great potential to individuals, that a smartphone can effectively replace all human actions and that the smartphone can simplify everyday life. For example an item was: “The smartphone simplifies my life.”
(3) Pervasiveness: it is a single item which measures opinions regarding the inevitability of technology, that is “It is unavoidable to use technological devices.”
(4) Sharing persuasive principle: in this case it is only one item that detects opinions about the idea that technology can change human behavior. The item was: “I don’t think that technology use can change our behaviors.”

The first 44 items were common to all participants in the study. After the scales just described two different applications for smartphones or other mobile devices were presented and participants were asked to select one of them, imagining to use it and respond to the subsequent questions. The applications were both persuasive, but the main difference relates to the direct and indirect benefits deriving from the use; ViviEco application implements direct benefits for the individual user and indirect for the community, while the use of PosiLive only benefits the individual user.

The mock applications were created to visually describe their operation, representing the display of a smartphone so that it would be clear to those who are accustomed to the use of this device. ViviEco was described as an application aimed to promote sustainable life choices while respecting the environment and others, it could provide advice and information to change some of the users’ daily habits.

**FIGURE 1:**
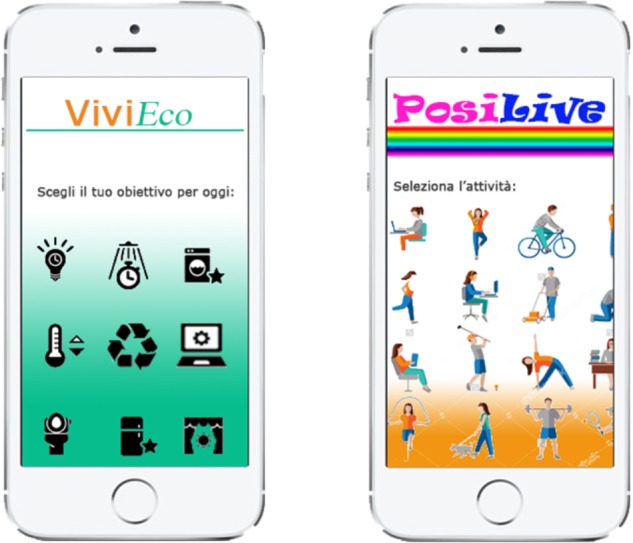
A sample screen for ViviEco (left) and PosiLive (right) mobile apps: different icons correspond to different activities.

The description given for the PosiLive application was different given its objectives: using PosiLive the user is helped in leading a healthy lifestyle by monitoring sports activities. It is possible to see a sample screen used for ViviEco and PosiLive (**Figure 1**).

The items following the presentations of the applications were partly taken from a similar research conducted in the field of persuasive applications for health (six overall). Halko and Kientz (2010) developed a study to assess the link between personality characteristics and persuasive properties of mobile applications created for the promotion of health. They used the Big Five Inventory as instrument to detect personality traits and they developed an application simulation to later evaluate users’ opinions (Halko and Kientz, 2010). The value of the study lies in the idea of being able to create applications designed on the needs of different personalities, in this way these technologies could achieve greater success.

The six questions prepared by Halko and Kientz investigate on a five-point Likert scale participants’ opinions on six different aspects of persuasive technologies: the fun, the probability of use in the future, the benefits to health, improvements on the quality of life, ease of use, time-saving. In addition to these six items other participants’ opinions about relevant dimensions of persuasive technologies were recorded:

(1) The persuasive power of the application. Four items were designed to assess the opinions related to some possible effects deriving from the use of the application: changing of behavior, having the motivational push to change habits, perceiving the smartphone as an effective tool for the transmission of relevant information, perceiving the suggestions received as the personal inner voice.
(2) The application informational power. Two items detected opinions regarding the idea that information received through the application are more relevant and reliable compared to other from online sources.

An additional item was added just for ViviEco to detect the perception of actions’ awareness, that is to believe that even the action of the individual through the application can have an actual impact on the environment.

Finally, there was an item (defined as fake test) that could serve as an indicator for possible falsified or untrusted compilations: participants were asked to enter their response on “completely agree.”

## Materials and Methods

### Participants

One hundred and eighteen participants (55 men, 63 women, *M*_age_ = 27.4, *SD* = 12.9) completed the study through the Google form questionnaire. Thirty seven participants were eliminated because they did not pass the fake test.

Participants were recruited through various ways: a link to the questionnaire was announced online to students of the University “G. d’Annunzio”. Leaflets with a brief description of the study were distributed, the invitation to participate and the link to the questionnaire were advertised at gyms in the towns of Chieti and Pescara and the metropolitan area. Finally, the invitation to participate was published on social networks, such as LinkedIn, Twitter, etc.

The sample was divided into men and women (**Table 1**). Other descriptive statistics were performed with regard to the preference of application, experiencing a high prevalence for ViviEco (**Table 2**).

**Table 1:** Descriptive statistics related to genre.

	Frequency	Percent	Valid Percent	Cumulative Percent
Male	55	46,6	46,6	46,6
Female	63	53,4	53,4	100
Total	118	100	100	

**Table 2:** Descriptive statistics relative to application preference.

		Frequency	Percent	Valid	Cumulative
				Percent	Percent
Valid	*ViviEco*	79	66,9	67,5	67,5
	*PosiLive*	38	32,2	32,5	100
	Total	117	99,2	100	
					
Missing	System	1	0,8		
	Total	118	100		

Subsequently, an analysis of the Chi square (χ^2^) was carried out to explore the different intersections of gender and application preference variables, specifically investigating if men and women had shown a different preference for the application purposes (sustainability and intervention in favor of the community vs. individual health and well-being), and if there was an effect of age in the preference of the applications.

With regards to age the sample was divided into three groups according to year of birth. The literature has suggested this because: some researchers have a found generational differences in the use of information and communication technologies (Lee and Coughlin, 2015). Consequently, the age variable was recoded in the following categories:

(1) *Baby boomers*, those born between 1945 and 1964;
(2) *Generation-X* refers to those born between 1963 and 1980;
(3) *Millennials*, those born between 1980 and 2000.

It must be specified that the test was not reliable because not all cells had at least five subjects; nevertheless it was interesting to underline the trend of no significance since in the three groups there was a similar relationship between choices, ViviEco was the application that received majority of the vote. This data therefore suggests that there is no link between gender, age and application preference.

## Results

After checking the quality distribution of the different scales (**Table 3**), which is good for all, except in the “video games” scale, which it was therefore excluded from the analysis, further analyses were conducted to verify the hypotheses.

**Table 3:** Reliability scales’ indexes.

Scale	Item number	Item example	Cronbach α	Mean	D.S.
Smartphone usage	9	How often do you read email on the smartphone	0,91	41,2532	16,24911
Videogame	9	How often do you play online with other people on the computer	0,91	12,9545	14,91998
Positive attitudes	6	I think it is important to be able to access Internet every time I want	0,851	21,513	4,77909
Negative attitudes	3	New technologies make life more complicated	0,66	8,2987	2,62997
Anxiety/dependence	3	I am dependent on my own technology	0,876	8,1818	3,22866
Technophobia	3	Technology deprive us from our freedom	0,632	8,9545	2,74717
Technophilia	3	The smartphone make my life easier	0,462	9,2403	1,96391
Persuasive power	4	I think that without this application I would not have the motivation needed to really change my habits	0,69	2,474	0,87221
Informative power	4	I think that information provided by the application are more reliable than other online information	0,497	3,039	0,74433

In order to test the first hypothesis (the intention to use the application in the future is influenced by: (a) the positive attitudes toward technology; (b) the persuasive power of the application; (c) the perceived fun) a linear regression was performed to predict the probability of use (the intention to use the application) on the basis of three predictors: positive attitudes toward technology, the persuasive power and the perceived fun. Preliminary analyses were performed to verify that there were no violations of the normality assumptions: linearity and multicollinearity. A significant regression was found [*F*(3.114) = 9.78; *p* < 0.001], with *R*^2^ = 0.20 (**Table 4**).

**Table 4:** Variance analysis to test the first hypothesis.

	ANOVA^a^				
	Sum of squares	df	Mean square	*F*	Sig.
Regression	15,17	3	5,057	9,788	0,000^b^
Residual	58,898	114	0,517	
Total	74,068	117		


*^a^Dependent variable: usage.*

*^b^Predictors: (constant), AttPOS, persuasive power, fun.*

The positive attitude was not significant as a predictor and for this reason only the other two variables were analyzed. This initial analysis make the first hypothesis partially verified and it also confirms the idea that an application is positively evaluated on the basis of the playful elements it contains. The strongest dimension of the persuasive power was related to the motivational incentive; according to the literature, there is a lack of motivation, especially for pro-environmental behaviors (Stern, 2000).

Subsequently, a mediation model was built to verify the second (persuasive power can have a mediating role in the relationship between the informational power and propensity to use an application) and the third hypothesis (persuasive power can have a mediating role in the relationship between perceived enjoyment and tendency to use the application).

There are two different ways to perform mediation analysis: multiple regression and structural equation modeling (SEM – Structural Equations Models). Some authors prefer to use the SEM method because it gives more control on the measurement error and provides reliable information on the suitability of the model (Baron and Kenny, 1986), in this case, the guidelines of Frazier et al. (2004) were followed, they recommended using multiple regression in case of a reduced sample.

In order to perform the mediation analysis, a SPSS function called PROCESS developed by Preacher et al. (2007) was used: incorporating the traditional approach (for example the Sobel test), the bootstrap approach and the approach developed by Baron and Kenny to quantify the indirect effects of the predictor on the dependent variable (Baron and Kenny, 1986). In this study the use of confidence intervals of the bootstrap method was necessary to avoid problems relating to the limited size of the sample, as already highlighted by Preacher et al. (2007).

**FIGURE 2:**
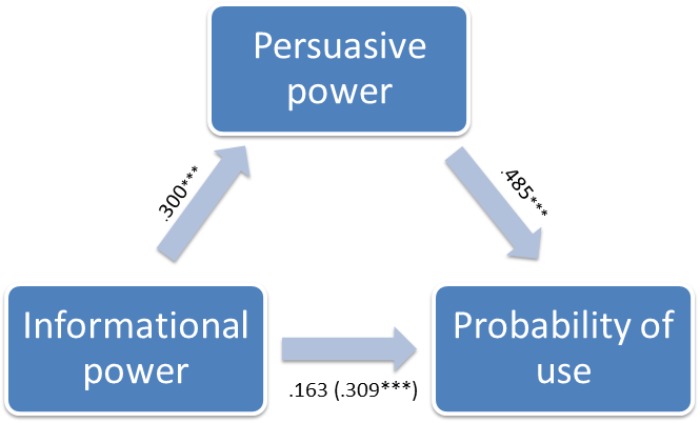
The first mediation analysis.

The first mediation analysis included the persuasive power as a mediator of the relationship between the informational power and the likelihood of future use. In particular it was found that the persuasive power mediates significantly the above relationship (indirect effect = 0.471, *SE* = 0.906, CI = 0.221); since the zero is not included in the confidence interval we can conclude that the indirect effect is significantly different from zero with an error probability of *p* < 0.05. So a change in the perception of the persuasive power modifies the influence of the informational power on the future probability of use with a mediation pattern that we call total (**Figure 2**).

The second mediation model considers that the persuasive power acts as a mediator in the relationship between the perceived fun and the probability of use, also in this case the mediation is significant (indirect effect = 0.206, *SE* = 0.103, CI = 0.043–0.452) with an error probability *p* < 0.05.

Finally an analysis of the potential impact of the application was performed through ANOVA, in order to test the fourth hypothesis (the perceived impact of the application ViviEco influences its persuasive power).

The ANOVA test detected a difference between the generations in the perceived impact (**Table 5**): this variable was integrated just for the application ViviEco and it refers in particular to the users’ awareness of their actions in the environment.

**Table 5:** Analysis of variance related to the difference between generations in the perceived impact.

	Impact				
	Sum of squares	df	Mean square	*F*	Sig.
Between groups	8,282	2	4,141	3,811	0,026
Within groups	83,668	77	1,087		
Total	91,95	79			

There is a significant difference between groups, specifically the mean of the baby boomers category which deviates moderately from the others (**Table 6**), on the basis of *post hoc* tests applied. This means that older people in the sample tended to have greater confidence in the fact that the action of the individual through the application could have a real impact on the environment. This information could be considered daunting by some as the majority of users of mobile applications are the younger ones. On the other hand, it is an indicator that definitely requires further investigation in the future: this can be due to a lack of trust in the information provided by the application or it can be due to a lack of education related to preservation and respect for the environment.

**Table 6:** Descriptive indicators of impact variable on the generation.

	Impact							
	*N*	Mean	Standard deviation	Standard error	95% Confidence Interval for mean		Minimum	Maximum
					Lower bound	Upper bound		
Baby boomers	6	4,3333	0,8165	0,33333	3,4765	5,1902	3	5
X-generation	10	3,3	0,67495	0,21344	2,8172	3,7828	2	4
Millennials	64	3,1094	1,10003	0,1375	2,8346	3,3842	1	5
								
Total	80	3,225	1,07885	0,12062	2,9849	3,4651	1	5

**FIGURE 3:**
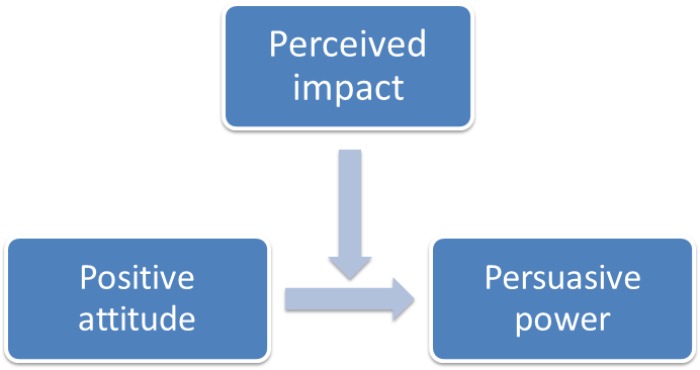
The moderation model.

In order to assess the link between the perceived impact and the persuasive power of the application ViviEco, a moderation model was created in which the individual perception of the impact moderates the relationship between positive attitudes toward technology and persuasive power, as shown in **Figure 3**.

The moderation is significant only for the high levels on the impact variable (**Table 7**), meaning that only those who express positive attitudes toward technology and believe a strong impact of their actions perceive a greater persuasive power of the application; this relation is not as significant for people who express moderate or low judgments on the impact.

**Table 7:** Detailed statistics related to the moderation model.

	Model				
	Coeff	*SE*	*t*	*p*	LLCI	ULCI
Constant	3,0649	0,0725	42,2894	0	2,9206	-3,2093
Impact	0,1517	0,0676	2,2435	0,0278	0,017	0,2864
POSattit	0,1289	0,0952	1,3533	0,18	-0,0608	0,3185
int_1	0,2601	0,0846	3,0756	0,0029	0,0917	0,428

This was particularly significant with regard to the collective benefits deriving from mobile technologies use, as can be inferred from the literature, the use of persuasive technology is influenced by several factors, most of personal derivation. For example, technology acceptance, motivational drive, consistency in the use of a device and in accomplishing a certain goal. In this case, the actions’ impact on the environment, that produces an indirect effect on the persuasive power of technology, calls into question a central element of our study: users will tend to use the application as much as they will perceive a collective benefit as well.

## Discussion

This study has both limitations and strengths: being an online survey there was the benefit from time and costs savings as well as the reduction of transcription errors, since the data was automatically entered into the database for later analysis, so the phase of manual transcription was avoided (Gabbiadini et al., 2011).

It is necessary to refer to experimental control processes, which are the set of procedures to limit the variability sources of the research itself: the control related to the questions presentation order has been secured; a Google Drive function in fact allows the random presentation of questions within each single scale. A single question in the questionnaire was inserted to identify any compilations unreliable or falsified: the so-called fake test. The detected number of fake compilations was relatively high: 36 compilations in a total of 154. For this reason, it can be planned a further investigation to be carried out with different tools, through the traditional paper and pencil method or having the opportunity to realize the applications and test it in a more controlled laboratory experiment.

The present study was limited to the investigation of attitudes and intentions, behaviors could not be measured since the applications were just introduced and participants had to imagine the usage. Compared to previous studies, the main difference concerns the consideration of the informational power as central variable in the intention to use the system and the evaluation of perceived collective benefits.

According to Venkatesh et al. (2003, p. 455), “attitudes toward using technology is defined as an individual’s overall affective reaction to using a system,” many different studies in line with this definition have been conducted in order to evaluate the intention to use a specific technology.

In several cases (Moses et al., 2014; Sarabadani et al., 2017) the perceived ease of use and the perceived enjoyment are the variables most effective in predicting the intention of use and the informational power is not considerate in past studies.

There have been also researches aimed at evaluating a match between personality traits and antecedents of technology use (Halko and Kientz, 2010; Godoe and Johansen, 2012; Orji et al., 2014), this could be further investigated in future developments of this research. Indeed, the application choice in our study (ViviEco or PosiLive) could be affected by some uncontrolled variables, both personal and contextual: for example, the personal relevance of the arguments, the predisposition to physical activity, the fact that winter season could discourage intentions related to outdoors physical activity, sensitivity to environmental issues and the sense of belonging toward the living environment. With regard to the latter aspect, Lalli (1992) demonstrated that people most identified with the places where they live are also more sensitive to environmental issues (Lalli, 1992).

The gender analysis that was not significant was carried out because of some evidences in psychosocial area: past studies have shown a gender gap with regard to the behaviors in the use of technology. For example, in the choice of online activities men are more inclined to amusement and fun, while women prefer to use the internet for communication or information purposes (Weiser, 2000). Contrary to expectations, there was no decisive propensity; in this regard it may be appropriate to investigate from the perspective of the Human Computer Interaction what actually leads men and women to use several of these specific technologies, and one might wonder if the gender-oriented applications are really necessary, which are dedicated explicitly to just one genre.

### Limitations

Overall the research work had inevitable limitations, which is worth mentioning for future research studies.

A problem encountered in the execution of this study concerned the low representativeness of the sample. Although gender was well distributed, age was not, there was a large number of young people and adolescents, who fall into the category millennials. Future researches could focus on obtaining a more representative sample. Some authors already analyzed the sampling problem of online surveys (Gosling et al., 2004): it seems that not all segments of the target population have the same access to the Internet and certain groups of people may have greater familiarity with technology and therefore are more likely to be part of the study sample (Suarez-Balcazar et al., 2009). On the contrary, some authors define this bias as a pure preconception, with evidences confirming that online samples can be as representative as traditional samples (Gosling et al., 2004).

Finally, it is necessary to emphasize that there is currently no pro-environmental application in the Italian market. In the future, studies should include an evaluation of the ethical dimension of such technology.

## Conclusion

The proposed investigation of the present research work is part of a multidisciplinary field, there are in fact soft disciplines involved such as social psychology and sociology, and technical disciplines belonging to the sphere of information technologies such as Human–Computer Interaction. The interest in persuasive technologies designed to change behavior is limited in the literature: the personal use of these technologies with social implications as well as exclusively personal and the relevance of informational power affecting the intention to use the technology.

Participants were asked to choose between two fictional applications and imagine using it: the first was meant to facilitate a healthy life style and stimulate physical activity, so it was able to cause personal advantages; the second application was meant to help the user make sustainable choices, in this way the application could promote benefits for the individuals and their community through environmental safeguarding. Participants were then asked to evaluate the application about the persuasive and the informative power.

It was interesting that older users were more convinced to use the application when they perceived they would generate a collective benefit. Current literature suggests that persuasive technology adoption is determined by personal factors such as motivational boosts or the perseverance in the tool usage; in this case the dimension related to altruism has a determinant role. This could be the first significant result: the intention to use the application is positively affected by the perceived environmental benefit. Even if the application was not properly tested, this can be a good premise for future experimental studies. Furthermore the generational gap is an unexpected dimension, since according to our results, the baby boomers are more aware of the indirect benefit on the environment, confirming that there is not a substantial difference with the so-called digital natives.

The second relevant result is related to the informational power: at the best of our knowledge this is the first study that considers this variable as a determinant in affecting the intention to use a technology that is not a health or medical technology.

In conclusion the contribution of the present study is related to the consideration of variables that are not yet well analyzed in literature: the informational power and the perceived collective benefit. We reckon that our results could be the premise for a model test with structural equation modeling.

Although more in-depth analyses are needed, the originality of this research lies in the proposed angle of analysis on persuasive technologies; therefore, new scenarios are open, in particular the sociological and psychosocial fields, investigating the effects that the personal use of a mobile device can produce on society. Based on our result, the question that arises concerns the ethical dimension of persuasive technology: what kind of information is suitable for the purpose of a mobile application?

## Author Contributions

SF and MC substantially contributed to the conception and design of the study, data collection, analysis and interpretation of data for the research. Both have written the manuscript and reviewed all parts of the manuscript and agree to be accountable for all aspects of the work.

## Conflict of Interest Statement

The authors declare that the research was conducted in the absence of any commercial or financial relationships that could be construed as a potential conflict of interest.

## Footnotes

1 Participants are asked to save an island from rising sea level, for each real pro-environmental activity which they register on the application, the virtual island situation would improve.
